# Supplementary material for: Netcombin: An algorithm for constructing optimal phylogenetic network from rooted triplets
Source: PLoS One. 2020 Sep 18;15(9):e0227842. doi: 10.1371/journal.pone.0227842 (PMC7500971; doi:10.1371/journal.pone.0227842)
Supplement: S4 File — (ZIP) [file pone.0227842.s004.zip › SequenceData-Triplet/sequenceData-10-20-30-0-Triplet/length 200, numseq 30, rate 10^-7/2 seq.pdf]

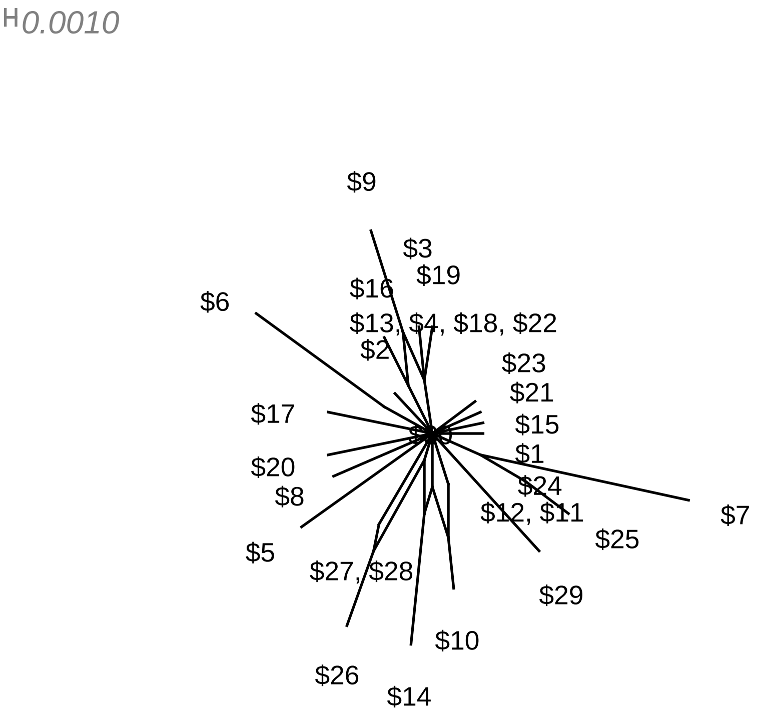
0.0010
$9
$3
$19
$16
$6
$13, $4, $18, $22
$2
$23
$21
$17
$15
$1
$20
$24
$8
$12, $11
$7
$25
$5
$27, $28
$29
$10
$26
$14
